# Open questions: development of tumor cell heterogeneity and its implications for cancer treatment

**DOI:** 10.1186/1741-7007-12-15

**Published:** 2014-03-05

**Authors:** Lukas Sommer

**Affiliations:** 1 Cell and Developmental Biology, University of Zurich, Institute of Anatomy, Winterthurerstrasse 190, CH-8057 Zurich, Switzerland

## Cellular and genetic tumor heterogeneity

The era of personalized medicine has been launched, with all its promises and hopes. Take cancer, for instance, where deep sequencing will make it possible to assess virtually all genomic alterations in a patient’s tumor tissue, and this at affordable costs. Already now, genomic testing can be used to predict whether or not a breast or colon cancer patient might benefit from a given treatment or whether the tumor of a melanoma patient carries a pathway mutation making it susceptible to specific pharmacological inhibitors. However, certain tumors such as melanoma are characterized by an abundance of mutations, and it is very demanding to determine which of these mutations are driver (providing a selective advantage to a tumor cell) or passenger (having no biological implication) mutations [1,2]. In many cancers, an astonishing inter- as well as intra-tumor heterogeneity makes it even more challenging to define prognostic signatures and potential therapies. Not only is the tumor of one patient different from the tumor of another patient, but also primary and metastatic lesions from different sites in the same patient exhibit vast genomic variations [3]. Even within a given biopsy there is cellular and genetic heterogeneity, which is likely associated with cellular differences in proliferation, survival mechanisms, invasiveness, and drug resistance [4]. This might explain why in melanoma, for instance, current targeted treatments only marginally improve perspectives for patients.

## Formation of tumor heterogeneity: learning from developmental biology

How heterogeneity is established within a tumor is of relevance, because knowledge of this process might inform about alternative treatment strategies. One model involves cancer stem cells that - like normal stem cells - are able to self-renew (thus contributing to tumor propagation) and to generate cellular heterogeneity by multi-lineage differentiation. Alternatively, tumor heterogeneity might arise by clonal evolution, driven by mutations that confer growth advantage of a cell and its clonal progeny over other cells. Possibly, these models are not mutually exclusive: for instance, a driver mutation might have to occur in a cell with stem cell properties in order for it to elicit its driving force. Or a mutation might promote cancer ‘stemness’ by reprogramming a cell that lacked this potential before. In any case, cellular heterogeneity existing prior to treatment could lead to resistance, in that intrinsic differences between different tumor cell fractions may influence drug responsiveness and promote selective growth, for example, of cancer stem cells or of cells with a distinct mutation signature. Thus, it is important to identify intrinsic properties characteristic for the various tumor cell fractions and, optimally, to find features common to all cell lineages making up a tumor.

Intrinsic properties of a tumor cell are conceivably determined by the cell’s developmental origin and cell lineage relationships. Indeed, tumors often share cellular and molecular features with cells of the tissue from which the tumor derived. In particular, there is increasing evidence that the developmental program of the tissue of origin is replayed during tumorigenesis. Melanoma, as an example, results from transformation of cells of the melanocyte lineage, which during embryonic development arises from neural crest cells. Intriguingly, it has recently been demonstrated that mechanisms underlying embryonic neural crest cell and melanocyte development also control melanoma formation *in vivo*[5]. In this study, the neural crest stem cell factor SOX10 was shown to be highly up-regulated in both murine and human melanoma cells; decreasing its expression levels prevented tumorigenesis without affecting normal melanocyte stem cell function. Likewise, the small Rho GTPase Rac1 is a factor known to be required for self-renewal and expansion of postmigratory neural crest stem cells [6], and recently, an activating mutation in *RAC1* has been identified to drive melanoma formation [7]. Thus we might be guided by developmental biology for the detection of novel players in tumor biology and hence to potential tumor therapy targets.

## Influences of environmental factors on intra-tumor heterogeneity

In addition to genetics, epigenetic mechanisms are crucial players in tumorigenesis. Because of their reversibility, epigenetic modifications likely allow dynamic switching from a silent state to growth and invasiveness, respectively, and could thus also contribute to intra-tumor heterogeneity or differences between primary tumors and their metastases. Such epigenetic modifications may be imposed on a tumor cell by microenvironmental pressure, such as provided by changes in oxygen levels, metabolites, or interactions with non-tumor cells. In addition, dynamic epigenetic changes are possibly a consequence of pharmacological drug exposure, which could be associated with resistance acquired during treatment. For instance, pharmacological inhibition of a given pathway might be circumvented in a cell by epigenetic de-repression of a novel compensatory pathway. In melanoma, dynamic changes of cell behavior have been observed in cell culture and *in vivo* upon exposure to inflammatory factors such as tumor necrosis factor-α [8]. Whether these changes indeed involve epigenetic reprogramming rather than selection of specific cell fractions is an open question that remains to be addressed, ultimately by single cell tracing in an *in vivo* context.

All in all, the various mechanisms possibly leading to intra-tumor heterogeneity ask for combinatorial treatment strategies. These may consist in targeting specific pathways together with inhibition of more general processes, such as those involving epigenetic control mechanisms. Ultimately, the clinics will tell [9] whether this approach will be successful in eradicating all tumorigenic cells in a patient - or at least in keeping them in check.
